# On-Ice Measures of External Load in Relation to Match Outcome in Elite Female Ice Hockey

**DOI:** 10.3390/sports7070173

**Published:** 2019-07-16

**Authors:** Adam Douglas, Kathryn Johnston, Joseph Baker, Michael A. Rotondi, Veronica K. Jamnik, Alison K. Macpherson

**Affiliations:** 1 Department of Kinesiology, York University, Toronto, ON M3J 1P3, Canada; 2 Catapult Sports, Melbourne, VIC 3181, Australia

**Keywords:** monitoring, PlayerLoad, skating load, explosive efforts, explosive ratio, percentage of high force strides

## Abstract

The aim of this study is to investigate the differences between select on-ice measures using inertial movement sensors based on match outcome, and to determine changes in player movements across three periods of play. Data were collected during one season of competition in elite female ice hockey players (N = 20). Two-factor mixed effects ANOVAs for each skating position were performed to investigate the differences in match outcome, as well as differences in external load measures during the course of a match. For match outcome, there was a small difference for forwards in explosive ratio (*p* = 0.02, ES = 0.26) and percentage high force strides (*p* = 0.04, ES = 0.50). When viewed across three periods of a match, moderate differences were found in skating load (*p* = 0.01, ES = 0.75), explosive efforts (*p* = 0.04, ES = 0.63), and explosive ratio (*p* = 0.002, ES = 0.87) for forwards, and in PlayerLoad (*p* = 0.01, ES = 0.70), explosive efforts (*p* = 0.04, ES = 0.63), and explosive ratio (*p* = 0.01, ES = 0.70) for defense. When examining the relevance to match outcome, external load measures associated with intensity appear to be an important factor among forwards. These results may be helpful for coaches and sport scientists when making decisions pertaining to training and competition strategies.

## 1. Introduction

Ice hockey is a major international sport with over one million registered participants across the globe [1]. For optimal performance, ice hockey athletes require well-rounded physical and physiological capabilities (amongst other qualities), including high aerobic and anaerobic capabilities, muscular strength, power, and endurance [2]. These physiological qualities are expressed in ice hockey by combining dynamic skating movement patterns with skills such as skating, shooting, and passing of the puck [1].

The incorporation of evidence-based approaches into training has become a critical component in many competitive sports, including ice hockey. This movement is reflected in the integration of sport science experts (analysts, medical teams, and researchers), as well as in an increased use of technology to help increase scientific rigor [3,4]. Specifically, the inclusion of wearable technologies (also known as ‘wearables’), such as heart rate monitors, global positioning systems (GPS), and accelerometers, has become common in many elite sport programs. In 2016, wearables (both consumer-based and athlete-based) were estimated to be a six-billion-dollar industry [5]. It is believed that the use of wearables may enhance coaches’ decision-making practices, while also helping to optimize player performance [6]. Specifically, training interventions, tactical assessments, competition preparation, and athlete feedback are just some of the areas influenced by the incorporation of wearable technologies in sport programs [7,8,9]. It is the hope that collecting and analyzing data from wearables, along with appropriately interpreting and applying the findings, can improve consistency of performance outcomes and the prevention of excess fatigue and overuse injuries [10]. The prevailing method to measure work performed on the ice in hockey research is through time-motion analysis (TMA). TMA has been widely used across many team sports [11,12,13], but is often criticized when applied to sports where player movements are extremely explosive and short in duration, and therefore difficult to record accurately [1].

The inclusion of wearables that measure external load variables in competitive sport programming may be an avenue for coaches and sport medicine practitioners, researchers, and strength and conditioning coaches to meaningfully track athletes’ performance in a way that extends beyond internal load methods—like subjective perceived ratings of exertion and heart rate measurement. External load refers to the interaction of volume and intensity that athletes experience during their sport, and often refers to the work performed by an athlete [14]. Typically, the quantification of this ‘work’ (i.e., movement demands) is captured through GPS, accelerometers, and/or video analysis. To date, the precision, reliability, and accessibility of GPS and accelerometers continue to improve, which has allowed sport science practitioners to use them at the highest levels of sport. For example, in 2015, the Federation Internationale de Football Association (FIFA) for men’s and women’s soccer allowed the collection of data during competitive matches using GPS [8]. Similarly, elite rugby league players wore GPS to capture physical demands during competitive matches [15]. Despite its prevalence in other team sports, the adoption of wearables by key decision-makers in ice hockey, including managers, coaches, and players, has been less immediate. This may be related to the limited empirical research on the degree of transferability between playing surfaces (i.e., ground compared to ice). Additionally, there are other administrative, financial, and logistical constraints that likely play a role in this slow uptake and implementation in the sport of ice hockey.

In the evolving climate of sport, the question of which statistics should be used has become a more important question than whether statistics should be used when it comes to many facets of decision-making. In the context of wearables, selecting which metrics to use has become a critical question. The value lies in the determination and prioritization of the key metrics for each sport that yield the most information, value, validity, reliability, and predictive capabilities. For example, Gabbett highlighted that valuable information was gleaned by comparing match data from wins and losses in team sports [15]. Specifically, the physical demands in elite rugby, measured using GPS and accelerometers, were higher when the team was winning versus losing, and when competing against lower ranked teams [15]. Although this study focused on rugby, the findings suggest that success in matches is linked to the team’s ability to maintain a higher playing intensity and may also be applicable to the sport of ice hockey. Similar findings in other team sports support the notion that player output varies depending on the result of the match. In soccer, it has been shown that high-intensity activity by key positions had a positive impact on winning [16,17]. The timing of these high-intensity events in soccer has also been shown to have a relationship with winning, as teams who display higher peak and mean running speeds in the second half of the game have a greater likelihood of winning the match [18].

Findings such as these could have direct implications for practice and competition strategies. Even slight changes to tactical strategies, athlete workload, and performance outputs may play a vital role in the outcome of a competition (e.g., shift changes and in-game strategies). Match outcome and its relationship to physical and tactical performance has been widely studied in other team sports [14,17,18,19,20,21,22], allowing coaches and sport scientists to prepare more effective training and competition strategies to have a positive impact on performance outcome. The application of wearable technology in elite ice hockey is an area of potential growth in sport science, with recent work exploring the difference between external and internal metrics between training and competition. Differences were evident when comparing data between playing positions, with defense having lower outputs of PlayerLoad, PlayerLoad·min^−1^, Training Impulse (TRIMP), and explosive efforts compared to forwards [23]. For the sport of ice hockey, there remains a void in the literature examining the playing conditions for elite level teams. The integration of player tracking technology at all levels of the sport has the potential to modernize the landscape of hockey analytics. Specifically, the inter- and intra-player and positional differences within and between competitive matches appear to be under-represented in the literature. Therefore, the primary aim of this study is to examine differences captured by wearable technology through inertial movement sensors worn by athletes in ice hockey matches. The hypothesis is twofold; higher player movement and intensity plays a role in match outcome, as well as player movement and intensity decrease across the game.

## 2. Materials and Methods

### 2.1. Study Design

A mixed effects design was employed to investigate the differences in on-ice measures of external load during competition, and whether these measures differed based on the outcome of the game (i.e., win or loss) by period of play, and the interaction of match outcome and period. This study used a retrospective, secondary data analysis with on-ice metrics for all 20 athletes collected during Hockey Canada’s Senior National Women’s Team matches during the 2016–2017 season. The team participated in 26 matches, with an outcome of 13 wins and 13 losses. Data were averaged for each position and reported for all three periods of play to allow for a repeated measures design comparing the differences specifically between match periods.

### 2.2. Participants

Elite female ice-hockey players (age = 24.8 ± 3.5 years; height = 171.6 ± 6.1 cm; body mass = 71.1 ± 6.1 kg) who represented their country in exhibition and international matches participated in this study. Using Baker and colleague’s taxonomy [24], this sample of athletes would be considered ‘expert’ based on their highest level of competition at the international level. The sample consisted of 13 forwards and seven defensive players. Goalies were excluded from the analysis due to their unique movement characteristics. All subjects gave their written informed consent for inclusion before they participated in the study. The study was conducted in accordance with the Declaration of Helsinki and approved under research ethics protocols by the Human Participants Review subcommittee at York University, Toronto, Canada.

### 2.3. Procedure

Each athlete wore a trunk-mounted (strapped to chest) Catapult S5 unit (Catapult Sports, Melbourne, Australia; firmware version 7.27) in a monitor-specific vest worn tight against the body in compliance with the manufacturer’s guidelines. The integration of 100-Hz triaxial accelerometry (quantifies linear motion in all directions—acceleration and deceleration) with triaxial 100-Hz gyroscopes (sampled at 2000° per second to measure body angular motion and rotation) and 100-Hz triaxial magnetometers (measures direction and orientation of body position) allowed for the quantification of PlayerLoad during indoor activity [25,26]. The triaxial gyroscope and magnetometer functions are necessary in the aggregation of data from each specific axis in the mediolateral, vertical, and anteroposterior planes of motion to quantify PlayerLoad during dynamic multi-plane body movement [26]. Data from the Catapult S5 units were downloaded to a database maintained by the National Sports Organization. Catapult OpenField software (OpenField 1.17.0 Catapult Sports, Melbourne, Australia) was used for proprietary postprocessing. Previous studies have reported the Catapult Sports S5 to have an excellent intradevice reliability for PlayerLoad values, with most Coefficient of Variation (CV) values rating low (<1.0%) [26,27]. It has also been reported that Intraclass Correlation Coefficient (ICC) ranged from 0.8 to 1.0 for intradevice comparison [27].

### 2.4. External Load Metrics

A description of the metrics used in the analysis are:

PlayerLoad is the summation of accelerations across all movements, divided by 100 [27]. This was expressed as total load (arbitrary units (au)). It is calculated as:

(1) $$\mathrm{PlayerLoad}=\sqrt{\frac{{\left(a_{y1}-a_{y-1}\right)}^{2}+{\left(a_{x}-a_{x-1}\right)}^{2}+{\left(a_{z}-a_{z-1}\right)}^{2}}{100}}$$

where: *a_y_* = Anteriorposterior acceleration; *a_x_* = Mediolateral acceleration; *a_z_* = Vertical acceleration.

Skating load is the summation of all peak accelerations recorded during the skating stride. Skating load is derived from a proprietary algorithm to identify the hockey stride based off the accelerometer tracing, gyroscope, and magnetometer sensors. The corresponding resultant acceleration peaks are calculated and multiplied by the athlete’s mass. This was expressed as total load (arbitrary units (au)). It is calculated as:

(2) $$\mathrm{Skating}\;\mathrm{Load}=\left(\sqrt{{\left(a_{y}\right)}^{2}+{\left(a_{x}\right)}^{2}+{\left(a_{z}\right)}^{2}}\times \mathrm{Player}\;\mathrm{Mass}\right)/100$$

Explosive efforts is the frequenc y of how many explosive movements were performed. High-intensity movements included: Rapid accelerations and decelerations, high-intensity skating, rapid changes of direction (skating-based or body contact), and high-intensity shots made by the player. This count was derived from Inertial Movement Analysis (IMA) data. Once identified, the sum of the X, Y area was calculated and expressed as the event magnitude (m·s^−1^) [28]. Any identified movement that occurred at a rate greater than 2 m·s^−1^ in any direction was considered an explosive effort [23].

Explosive ratio is a ratio calculated by taking the total number of explosive efforts and dividing it by PlayerLoad. This provides information as to the athletes’ ability to produce explosive movements based off their total load accumulation throughout the course of a match.

Percentage high force strides captures the percentage of all the ice hockey strides that occurred in the high force band. For female ice hockey players, strides that exceed 140 au skating load are coded as high force strides based off banding recommendations from the manufacturer.

### 2.5. Statistical Analysis

For each variable listed above, a two-factor mixed effects ANOVA was performed to investigate the difference in matches that were won and lost, as well as the difference in variables across the three periods of play. A normal distribution of data was examined using the Shapiro-Wilk’s test and homogeneity of variance was confirmed with Levene’s test, which supported the use of parametric methods of analysis. Due to the differences in match demands, forwards and defensive players were analyzed separately. All data were processed in RStudio (version 1.0.153, R Core Team, Vienna, Austria). Differences between match outcome as well as between periods were analyzed using effect size (ES) statistics. To ensure consistency in reporting of results and comparability across all analyses, the partial eta-squared statistics from ANOVA were also converted to ES [29]. Effect sizes were categorized using the following descriptors: <0.2—trivial, 0.2–0.6—small, >0.6–1.2—moderate, >1.2–2.0—large, >2.0—very large [30]. Data are presented as mean ± SD and statistical significance was set at *p* ≤ 0.05.

## 3. Results

### 3.1. Descriptive Summary of On-Ice Metrics and Two-Way ANOVA Results

The descriptive statistics for the different metrics are summarized in Table 1 for forwards and Table 2 for defense. The results of the two-way mixed effect ANOVAs can be found in Table 3 for forwards and Table 4 for defense.

### 3.2. PlayerLoad

For the defensive players, there was no statistically significant difference between wins and losses, however, a statistically significant difference was found between periods (F(2,72) = 4.51, *p* = 0.01, ES = 0.70), with the first period having the highest PlayerLoad (482.58 ± 65.24), followed by the second period (444.04 ± 73.29) and third period (425.38 ± 94.40). Post hoc testing using Tukey HSD indicated there was a moderate decrease from the first period to third period (*p* = 0.01, ES = 0.70).

### 3.3. Skating Load

For forwards, a two-way ANOVA identified a statistically significant difference for skating load between periods (F(2,72) = 4.92, *p* = 0.01, ES = 0.75), with the first period having the highest load (522.56 ± 23.70), followed by the third period (491.86 ± 62.36), with the second period demonstrating the lowest load (480.1 ± 55.82). Post hoc testing using Tukey HSD indicated a moderate decrease from the first to second period (*p* = 0.01, ES = 0.98).

### 3.4. Explosive Efforts

There was a statistically significant difference for explosive efforts between periods for the forwards (F(2,72) = 3.35, *p* = 0.04, ES = 0.63), with the first period having the highest rating (1044.42 ± 129.06), followed by the third period (963.54 ± 161.25), and second period (938.00 ± 161.25). Post hoc testing using Tukey HSD indicated that there was a moderate decrease from the first period to second period (*p* = 0.04, ES = 0.73).

For defensive players, a statistically significant difference between periods was also found (F(2,72) = 3.45, *p* = 0.04, ES = 0.63), with the first period reporting the highest explosive efforts (482.58 ± 65.24), followed by the second period (444.04 ± 73.29) and the third (425.28 ± 94.40). Post hoc testing using Tukey HSD showed there was a moderate decrease between the first period and third period (*p* = 0.03, ES = 0.71).

### 3.5. Explosive Ratio

For forwards, there was a main effect of match outcome (F(1,72) = 5.30, *p* = 0.02, ES = 0.26), such that the average explosive ratio in matches that were won were higher (1.46 ± 0.10) than lost (1.41 ± 0.11). There was a statistically significant difference between periods (F(2,72) = 6.63, *p* = 0.002, ES = 0.87), with the first period demonstrating the highest explosive ratio (1.49 ± 0.10), followed by the second (1.41 ± 0.09) and the third (1.40 ± 0.12). Post hoc testing using Tukey HSD indicated a moderate decrease from the first period to both the second (*p* = 0.01, ES = 0.84) and third periods (*p* = 0.004, ES = 0.81).

For defensive players, there was a main effect of period number (F(2,72) = 4.51, *p* = 0.01, ES = 0.70), whereby the first period reported the highest explosive ratio (1.32 ± 0.12), followed by the second (1.26 ± 0.11) and third (1.21 ± 0.16). Post hoc testing using Tukey HSD showed a moderate decrease between the first period and third period (*p* = 0.01, ES = 0.78).

### 3.6. Percentage High Force Strides

The forward group showed a statistically significant difference between match outcome (F(1,72) = 4.21, *p* = 0.04, ES = 0.51), where a higher percentage of high force strides were found in matches that were won (17.27 ± 1.30) compared to matches that were lost (16.48 ± 1.98).

## 4. Discussion

In this study, we report data from wearable technology using selected metrics of external load collected during matches, and their differences based on match outcomes across three periods of play. Our results generally support the use of wearable technology for collecting player data related to volume and intensity, as measured through various metrics of external load. When examining the relevance to match outcome, indices of external load appears to be an important factor in this sample of elite female ice hockey players, but only among the forwards where a significant difference for explosive ratio and the percentage of high force strides was found in matches that were won versus lost. Both are indicators of on-ice skating intensity, suggesting that the ability to have a high output of skating intensity is important for success in matches.

The results also demonstrate a significant drop in external load measures from the first period to the second period. The second period had lower measured skating load and explosive efforts compared to the first and third period. With the sport of ice hockey being broken up into three 20-min periods interspersed with a 15-min intermission, one could surmise that the drop-off would be similar across the later periods due to the intermission, which affords the athletes the ability to rest and recover. Evidence of this declining output was seen in the defensive group, where significant differences in period output was found in PlayerLoad, explosive efforts, and explosive ratio. In all three of these variables, the first period had the highest output and the third period the lowest, which might be attributed to the accumulation of fatigue. The recent work by Lignell and colleagues [31] in men’s ice hockey using video-based external load monitoring supports evidence of declining outputs because of fatigue. The researchers showed that the average sprint-skating speed was lower in the later periods of the match. There may be several explanations as to why this occurs in ice hockey. One reason could be the inability to repeat the appropriate number of high-speed bouts within a hockey match. It has been shown in ice hockey players that a high aerobic power increases the ability to recover from repeated bouts of anaerobic power [32]. Peterson and colleagues found a high correlation between maximal oxygen uptake and fatigue during the later periods of a mock hockey match in high-level collegiate hockey players [33]. Another explanation could be attributed to the tactical situation of the match during the later phases of competition. If a team is winning, the team may adjust their strategy to play more conservatively, which could alter player output, unrelated to fitness or fatigue. In most high-level ice hockey, coaches prefer to play an assertive style forecheck when the score is close to attack the opponent and increase the pace of play. This up-tempo style relies on a fast-skating aggressive style of forecheck most coaches employ from a tactical perspective [34].

Another interesting finding from on-ice tracking data of men’s professional ice hockey reported players performed an average of seven high-intensity skating bouts every minute [31], which is proportionally much higher than reported in other field and court-based team sports [35,36,37]. According to the published TMA literature for female ice hockey, forwards had an average of 18 forward shifts per match, with a mean duration of 48 s; whilst their defensive teammates averaged 15 defensive shifts per match with an average shift duration of 43 s. Each shift consisted mainly of low- to moderate-intensity skating interspersed with brief, intermittent high-intensity bouts [11].

Additionally, other studies have assessed the physiological demands of ice hockey during competition. In conjunction with aerobic training, it has also been shown that a positive relationship exists between ice hockey players with higher anaerobic power scores and their draft position in the National Hockey League [38]. Both are related to the outputs required for individual on-ice success, (i.e., the ability to produce high-intensity output and to repeat the high-intensity bouts). The finding that positional differences relate to match outcome did not come as a surprise, as it has been reported that the match demands placed on forwards and defense are vastly different [23]. Female ice hockey forwards have been found to have greater anaerobic power output, as well as a higher aerobic capacity, when compared to female ice hockey defensive players [39], along with a higher duration and frequency of high-intensity skating than defensive players [11]. These differences are most likely attributed to the different positional demands, whereby the defensive group retreats more often and typically covers a lower proportion of the ice. Positional differences were also reflected in men’s professional ice hockey, with forwards exhibiting a higher average skating speed and covering a greater distance at high-intensity [31]. Taken together, it appears that for success in ice hockey, it is important for players to be able to tolerate high-intensity and high-velocity efforts, as well as the ability to endure repeat anaerobic bouts. This can have important implications for coaches and sport medicine practitioners alike to help inform periodized training and competition practices, especially as it relates to match outcomes.

To the authors’ knowledge, this is the first study of its kind to examine wearable technology during competitive matches in the sport of women’s ice hockey. Some studies have examined the physiological demands in relation to team success in ice hockey [40], however, this study is unique in that multiple performance metrics were assessed via wearable technology (PlayerLoad, skating load, explosive efforts, explosive ratio, and percentage high force strides). While our study is novel in terms of using measurable match data from accelerometers to uncover determinants of match play and match outcome, there are certain limitations that are important to acknowledge. The first is the length of time the data were collected. Increasing the study length to include multiple seasons with the same athlete sample and coaching staff could allow for patterns to emerge, both from the main effects and the interactions between winning and losing and player metrics during different periods of the game. A second limitation is that due to the variable nature of player deployment in ice hockey, large standard deviations were present. Player ice time is largely dictated by coaching strategy, and thus using a positional average dataset can be limiting. Further research could focus on the higher-performing players (e.g., top six forwards and top four defense). Furthermore, while the inclusion of high-level athletes allowed for a unique and valuable data set, it limits the generalizability of our results to other populations, such as a non-expert group of ice hockey players, which would allow researchers to track changes between levels of performance.

## 5. Conclusions

The results of the present study demonstrate the potential benefits of using wearable technology to collect data on performance metrics in the sport of ice hockey. With appropriate assessment and implementation, it may positively impact coaches’ decision-making as it pertains to game demands and game outcome. The results suggest the intensity measure of external load of game play by forwards has an impact on match outcome. Secondary results show between-period differences for forwards with the second period typically lower in external load measures than the first period or third period. For defensive players, a difference in external load across all three periods was evident, with the main findings suggesting a drop-off between the first and third periods. There is a paucity of research in the application of wearable technology to monitor external load in ice hockey, and as the body of research grows it will be important to understand the unique movement patterns and movements strategies that are inherent to ice hockey, such as the impact of low-intensity locomotion or gliding and its relationship to skating performance. Future research could further investigate the reasons behind the decreased output across a match to determine if it is physiological fitness- or fatigue-related, as well as explore the use of some of the between-period interventions utilized by other high-performance teams in other sports.

## Figures and Tables

**Table 1:** Descriptive statistics of select external load variables for forwards, by game outcome and period.

Select External Load Variable—Forwards			Mean	SD
PlayerLoad	Win	First Period	692.82	104.65
		Second Period	674.30	92.97
		Third Period	667.37	112.58
	Loss	First Period	716.04	112.00
		Second Period	659.79	134.08
		Third Period	714.52	139.19
Skating Load	Win	First Period	527.24	20.39
		Second Period	493.04	39.50
		Third Period	484.94	37.97
	Loss	First Period	517.88	26.57
		Second Period	467.11	67.58
		Third Period	498.78	80.97
Explosive Efforts	Win	First Period	1043.31	143.74
		Second Period	968.92	133.20
		Third Period	945.62	138.93
	Loss	First Period	1045.54	118.49
		Second Period	907.08	185.36
		Third Period	981.46	194.42
Explosive Ratio	Win	First Period	1.51	0.10
		Second Period	1.44	0.09
		Third Period	1.43	0.11
	Loss	First Period	1.47	0.10
		Second Period	1.38	0.07
		Third Period	1.37	0.13
Percentage High Force Strides	Win	First Period	17.70	1.21
		Second Period	17.11	1.24
		Third Period	17.00	1.43
	Loss	First Period	16.41	1.78
		Second Period	16.25	1.99
		Third Period	16.78	2.26

**Table 2:** Descriptive statistics of select external load variables for defense, by game outcome and period.

Select External Load Variable—Defense			Mean	SD
PlayerLoad	Win	First Period	363.19	60.04
		Second Period	358.43	58.29
		Third Period	346.64	65.55
	Loss	First Period	371.37	54.99
		Second Period	346.59	50.51
		Third Period	354.61	65.96
Skating Load	Win	First Period	477.76	48.48
		Second Period	453.19	52.43
		Third Period	434.41	49.62
	Loss	First Period	484.66	59.74
		Second Period	453.41	82.51
		Third Period	445.38	98.04
Explosive Efforts	Win	First Period	478.23	77.25
		Second Period	449.31	87.78
		Third Period	421.38	88.47
	Loss	First Period	486.92	53.47
		Second Period	438.77	58.53
		Third Period	429.38	103.46
Explosive Ratio	Win	First Period	1.32	0.11
		Second Period	1.25	0.13
		Third Period	1.21	0.13
	Loss	First Period	1.32	0.13
		Second Period	1.27	0.09
		Third Period	1.21	0.19
Percentage High Force Strides	Win	First Period	10.20	2.00
		Second Period	10.21	2.41
		Third Period	9.93	1.99
	Loss	First Period	10.45	2.40
		Second Period	10.55	2.39
		Third Period	10.49	2.44

**Table 3:** Two-way ANOVA results of select external load variables on game outcomes and periods for forwards.

Select External Load Variables—Forwards		F-Statistic	*p*	ES
PlayerLoad	WinLoss	F(1,72) = 0.48	0.48	0.20
	Period	F(2,72) = 0.51	0.51	0.29
	WinLoss:Period	F(2,72) = 0.63	0.63	0.20
Skating Load	WinLoss	F(1,72) = 0.39	0.53	0.20
	**Period**	**F(2,72) = 4.92**	**0.01**	**0.75**
	WinLoss:Period	F(2,72) = 1.02	0.37	0.35
Explosive Efforts	WinLoss	F(1,72) = 0.05	0.82	0.06
	**Period**	**F(2,72) = 3.35**	**0.04**	**0.63**
	WinLoss:Period	F(2,72) = 0.68	0.52	0.29
Explosive Ratio	**WinLoss**	**F(1,72) = 5.30**	**0.02**	**0.26**
	**Period**	**F(2,72) = 6.63**	**0.002**	**0.87**
	WinLoss:Period	F(2,72) = 0.10	0.91	0.02
Percentage High Force Strides	**WinLoss**	**F(1,72) = 4.21**	**0.04**	**0.50**
	Period	F(2.72) = 0.33	0.72	0.20
	WinLoss:Period	F(2,72) = 0.65	0.52	0.29

Note: Bold font indicates *p* < 0.05.

**Table 4:** Two-way ANOVA results of select external load variables on game outcomes and periods for defense.

Select External Load Variables—Defense		F-Statistic	*p*	ES
PlayerLoad	WinLoss	F(1,72) = 0.02	0.89	0.06
	**Period**	**F(2,72) = 4.51**	**0.01**	**0.70**
	WinLoss:Period	F(2,72) = 0.08	0.92	0.09
Skating Load	WinLoss	F(1,72) = 0.15	0.69	0.09
	Period	F(2,72) = 2.52	0.08	0.50
	WinLoss:Period	F(2,72) = 0.04	0.96	0.06
Explosive Efforts	WinLoss	F(1,72) = 0.01	0.91	0.06
	**Period**	**F(2,72) = 3.45**	**0.04**	**0.63**
	WinLoss:Period	F(2,72) = 0.12	0.89	0.11
Explosive Ratio	WinLoss	F(1,72) = 0.02	0.89	0.06
	**Period**	**F(2,72) = 4.51**	**0.01**	**0.70**
	WinLoss:Period	F(2,72) = 0.08	0.92	0.09
Percentage High Force Strides	WinLoss	F(1,72) = 0.55	0.46	0.20
	Period	F(2.72) = 0.04	0.96	0.06
	WinLoss:Period	F(2,72) = 0.03	0.97	0.06

Note: Bold font indicates *p* < 0.05.
